# Family Availability and Its Influence on Informal and Formal Care Used by Adults With Dementia in the United States

**DOI:** 10.1093/geroni/igaa057.1129

**Published:** 2020-12-16

**Authors:** HwaJung Choi, Michele Heisler, Tsai-Chin Cho, Cathleen Connell

**Affiliations:** 1 University of Michigan, Ann Arbor, Michigan, United States; 2 University of Michigan, Ann Arbor, United States

## Abstract

This research is to provide national estimates of spouse and adult child availability to care for adults 55+ with dementia and to examine associations between availability and formal and informal care utilization. Only 23% of adults with dementia had a non-disabled spouse; 66% had an adult child living less than 10 miles away. Substantial variations in family availability were discovered across demographic and socioeconomic groups. For example, 29% of non-Hispanic blacks vs. about 40% of other racial/ethnic groups (OR=0.63; p<0.001) had a spouse. Only 16% of the bottom wealth quartile had a spouse compared to 61% of the top quartile (OR=0.13; p<0.001). In contrast, the greater share of non-Hispanic blacks than non-Hispanic whites had a coresident adult child (OR=2.07; p<0.001) and a non-employed adult child (OR=1.45; p<0.001). Hispanics had the most family availability from both spouse and child. Having a spouse was significantly associated with a lower probability of receiving formal care; AOR=0.54 (95% CI 0.46-0.64) for any formal care; AOR=0.50 (95% CI 0.39-0.64) for institutional care. Having a coresident adult child in year T-2 also substantially reduced the probability of receiving formal care in year T (AOR=0.37; 95% CI 0.29-0.48). The presence of a spouse and co-resident adult child significantly reduce the use of formal care by adults with dementia in the US. Policies and interventions that rely on family members to provide dementia should reflect the substantial heterogeneity in potential family availability across racial/ethnic and socioeconomic groups.

